# Structures, Chemical Conversions, and Cytotoxicity of Tricholopardins C and D, Two *Tricholoma* Triterpenoids from the Wild Mushroom *Tricholoma pardinum*

**DOI:** 10.1007/s13659-021-00297-x

**Published:** 2021-01-15

**Authors:** Chen Shi, Yue-Ling Peng, Juan He, Zheng-Hui Li, Ji-Kai Liu, Tao Feng

**Affiliations:** 1 School of Pharmaceutical Sciences, South-Central University for Nationalities, Wuhan, 430074 People’s Republic of China; 2 The Third People’s Hospital of Datong, Datong, 037000 People’s Republic of China

**Keywords:** *Tricholoma pardinum*, *Tricholoma* triterpenoids, Chemical conversion, Cytotoxicity

## Abstract

Two undescribed *Tricholoma* triterpenoids, namely tricholopardins C (**1**) and D (**2**), were isolated from the wild mushroom *Tricholoma pardinum*. Their structures with absolute configurations were elucidated by spectroscopic methods, as well as the single crystal X-ray diffraction. Compounds **1** and **2** were further obtained by chemical conversions from the known analogues. Compound **1** showed significant cytotoxicity to MCF-7 and Hela cell lines with IC_50_ values of 4.7 μM and 9.7 μM, respectively. Its mechanism of inducing MCF-7 cell apoptosis was studied briefly.

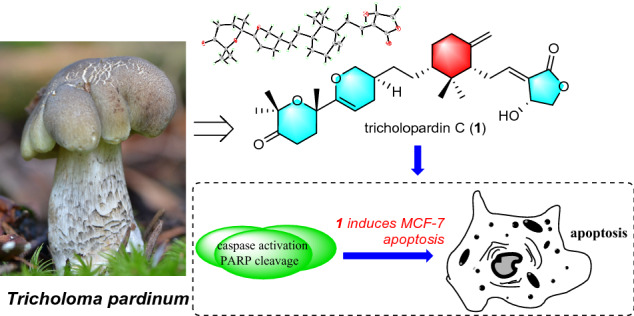

## Introduction

Cancer represents one of the most threats to human health, accounting for 13% of all deaths worldwide according to the World Health Organization [1]. Although a great number of chemotherapeutic drugs are available over the counter, the cancer cure rate is really difficult to be improved effectively [2]. Natural products remain an important resource for the identification of novel therapeutic leads and provide avenues for the discovery of new molecular targets [3], while fungal metabolites with a variety of chemical structures and diverse biological activities play an important role in drug discovery [4].

**Fig. 1:**
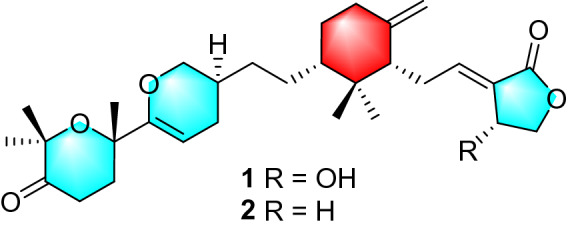
Structures of compounds **1** and **2**

*Tricholoma* triterpenoids (TTs) are a class of special natural products featuring a central methylenecyclohexane ring linked to a *γ*-lactone substituent and a pyrane ring shared a spiroketal carbon with the bridged heterocyclic system [5, 6]. So far, TTs have been found in a very narrow distribution limited to several mushrooms of the genus *Tricholoma* (Tricholomataceae), such as saponaceolides A‒G [6, 7] and T [8] from *T. saponaceum*, trichomycins A and B from a new species *Tricholoma* sp. AU1 [9], saponaceolides H‒S [10, 11] and terreolides A‒F [10] from *T. terreum*, and tricholopardins A and B from *T. pardinum* [5]. Previous studies have demonstrated that TTs have promising cytotoxicity and anti-inflammatory effects. Saponaceolides A and B were found to inhibit the growth of a human colon adenocarcinoma cell line (line LoVo) with ID_50_ values of 450 ng/mL and 163 ng/mL, respectively [6, 7] while saponaceolide Q showed cytotoxicity to four human cancer cell lines [11]. In addition, tricholopardin A potently inhibited nitric oxide production in lipopolysaccharide-induced RAW264.7 macrophages with an IC_50_ of 0.08 μM [5]. All the information suggested that TTs have a good research prospect. As our continuous search for new and bioactive metabolites from *Tricholoma* mushrooms [5, 10, 14], a further chemical investigation on the mushroom *T. pardinum* was undertaken. As a result, two novel TTs namely tricholopardins C (**1**) and D (**2**) were obtained (Fig. 1). Their structures were established by means of spectroscopic methods. Their absolute configurations were determined by the single crystal X-ray diffraction, as well as the chemical conversions from the known analogues. Both two compounds possessed a novel linear structure involving four independent rings. Compounds **1** and **2** were evaluated for their cytotoxicity against human breast adenocarcinoma cell line (MCF-7) and human cervical carcinoma cell lines (Hela). The mechanism of **1** inducing MCF-7 cell apoptosis was also investigated.

## Results and Discussion

A sample of air-dried fruiting bodies of *T. pardinum* (1.5 kg) was extracted using MeOH and partitioned using EtOAc to give an EtOAc extract (56 g). The latter was separated through column chromatography to afford **1** (1.2 mg) and **2** (2.6 mg). More samples of **1** (3.5 mg) and **2** (2.1 mg) were prepared by chemical conversion from saponaceolides A and B, respectively.

**Fig. 2:**
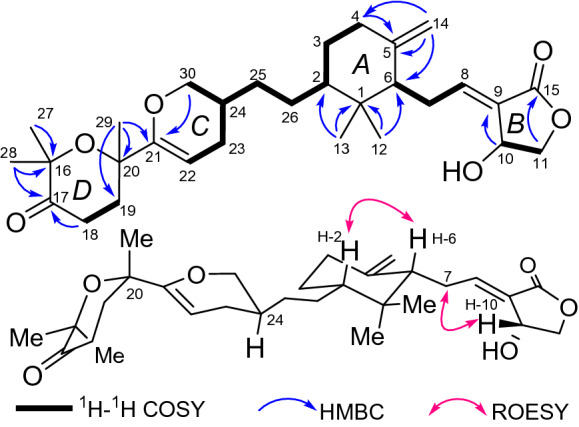
Key 2D NMR correlations for **1**

Tricholopardin C (**1**) was isolated as colorless crystals. Its molecular formula C_30_H_44_O_6_ was identified by HRESIMS data, corresponding to nine degrees of unsaturation. IR absorption bands at 3432 cm^‒1^ and 1736 cm^‒1^ revealed OH and C = O functional groups. In the ^1^H NMR spectrum, five singlets for methyl groups were readily identified. In addition, four olefinic protons including two singlets at *δ*_H_ 4.90 and 4.58 assigned for a terminal double bond were detected. The ^13^C NMR spectrum revealed thirty carbon resonances, which were assigned by DEPT and HSQC spectra into five CH_3_, eleven CH_2_, six CH, and eight C (Table 1). Preliminary analysis of these data, as well as the 2D NMR data (Fig. 2), suggested that **1** should be a TTs derivative. The existence of a central cyclohexane ring *A* linked to a *γ*-lactone *B*, identical to that of saponaceolide A, was established by comparison with data of saponaceolide A (Fig. 2). Analysis of ^1^H‒^1^H COSY data revealed a linkage of starting from an olefinic carbon C-22 to C-23, C-24, and C-30. In association with the key HMBC correlations from *δ*_H_ 4.01 (1H, m, H-30a) and 3.54 (1H, dd, *J* = 10.0, 8.8 Hz, H-30b) to *δ*_C_ 156.2 (s, C-21), a dihydropyran *C* was established. It was connected to ring *A* by two methylene carbons (CH_2_-25 and CH_2_-26) as established by the ^1^H‒^1^H COSY and HMBC data. In the HMBC spectrum, a methyl signal at *δ*_H_ 1.40 (3H, s, H_3_-29) showed three correlations to *δ*_C_ 74.5 (s, C-20), 30.0 (t, C-19), and C-21, while the other two methyl signals at *δ*_H_ 1.34 (3H, s, H_3_-28) and 1.36 (3H, s, H_3_-27) showed key correlations to *δ*_C_ 80.3 (s, C-16) and 214.6 (s, C-17). The information, as well as the MS data, suggested that the remaining eight carbons constructed a tetrahydropyran *D* involving the substituents of two methyls at C-16, one carbonyl carbon at C-17, and one methyl at C-20. Therefore, the planar structure of **1** was elucidated as shown.

**Table 1:** ^1^H (600 MHz) and ^13^C (150 MHz) NMR data for **1** (methanol-*d*_4_)

No	**1**		**2**	
	*δ*_H_	*δ*_C_	*δ*_H_	*δ*_C_
1		39.8, C		39.7, C
2	1.15, m	48.1, CH	1.14, m	48.2, CH
3a	1.81, m	30.1, CH_2_	1.80, m	30.2, CH_2_
3b	1.14, m		1.13, m	
4a	2.33, m	37.0, CH_2_	2.31, m	37.3, CH_2_
4b	1.95, m		1.94, m	
5		148.0, C		148.1, C
6	1.92, m	53.6, CH	1.93, m	53.5, CH
7a	2.61, m	26.2, CH_2_	2.41, m	26.9, CH_2_
7b			2.28, m	
8	6.99, t (6.9)	149.5, CH	6.72, t (6.8)	142.3, CH
9		127.9, C		124.6, C
10	5.05, m	66.3, CH	2.88, m	25.3, CH_2_
11a	4.44, dd (10.4, 6.0)	74.3, CH_2_	4.37, t (7.5)	65.4, CH_2_
11b	4.26, dd (10.4, 1.8)			
12	0.61, s	15.1, CH_3_	0.58, s	14.9, CH_3_
13	1.03, s	26.7, CH_3_	1.02, s	26.6, CH_3_
14a	4.90, s	108.3, CH_2_	4.84, s	107.7, CH_2_
14b	4.58, s		4.40, s	
15		169.9, C		171.5, C
16		80.3, C		80.3, C
17		214.6, C		214.6, C
18	2.43, m	33.3, CH_2_	2.43, m	33.3, CH_2_
19a	2.28, m	30.0, CH_2_	2.28, m	30.0, CH_2_
19b	1.99, m		2.04, m	
20		74.5, C		74.5, C
21		156.2, C		156.2, C
22	4.93, dd (4.3, 3.0)	94.9, CH	4.93, dd (4.4, 3.0)	94.9, CH
23a	2.17, m	27.3, CH_2_	2.17, m	27.3, CH_2_
23b	1.68, m		1.68, m	
24	1.75, m	32.5, CH	1.74, m	32.5, CH
25a	1.40, m	31.2, CH_2_	1.40, m	31.2, CH_2_
25b	1.10, m		1.10, m	
26a	1.59, m	28.0, CH_2_	1.59, m	28.1, CH_2_
26b	0.93, m		0.92, m	
27	1.36, s	27.9, CH_3_	1.36, s	27.9, CH_3_
28	1.34, s	26.2, CH_3_	1.34, s	26.2, CH_3_
29	1.40, s	28.3, CH_3_	1.40, s	28.3, CH_3_
30a	4.01, m	70.3, CH_2_	4.02, m	70.3, CH_2_
30b	3.54, dd (10.0, 8.8)		3.54, dd (10.1, 8.7)

**Scheme 1:**
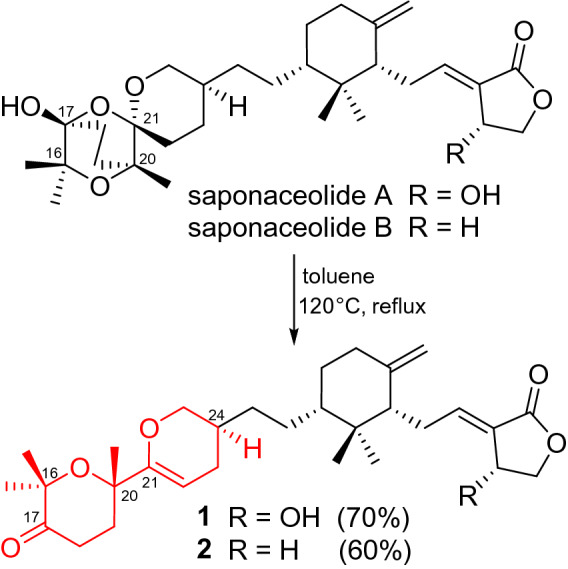
Chemical conversions of **1** and **2**

**Fig. 3:**
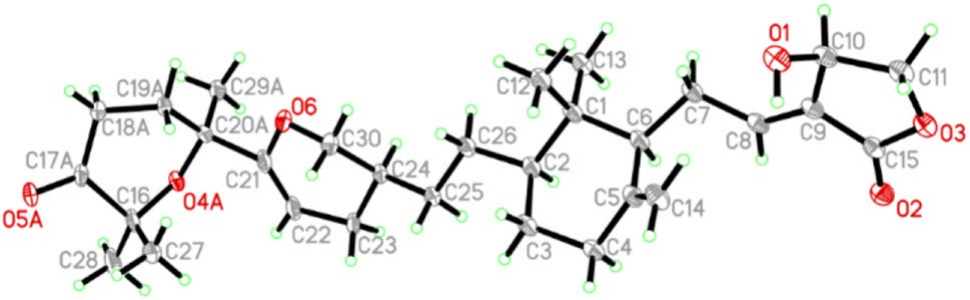
ORTEP diagram of **1** showing absolute configuration

In the ROESY spectrum, one cross peak of H-10/H-7 indicated the double bond between C-8 and C-9 to be *trans-*form, while a cross peak of H-2/H-6 indicated that H-2 and H-6 were on the same face (Fig. 2). However, the stereochemistry of C-20 and C-24 could not be elucidated by the ROESY data. The chemical conversion of **1** was achieved from saponaceolide A (Scheme 1), obtained from the same source [1], whose absolute configuration has been demonstrated previously [2, 8]. This conversion suggested the stereo configurations of C-20 and C-24 to be *S*. In addition, based on the sample accumulation by the chemical conversion, a single crystal for **1** was obtained from a mixture of methanol/H_2_O (20/1, v/v) after attempts, and the single crystal X-ray diffraction for **1** not only confirmed the planar structure as established by the spectroscopic methods but also determined the absolute configuration by the Flack parameter of −0.01(10) (CCDC: 1,907,789) (Fig. 3).

Tricholopardin D (**2**) was isolated as a colorless oil. The molecular formula C_30_H_44_O_5_ was revealed by the HRESIMS data, 16 mass units less than that of **1**. All the ^1^H and ^13^C NMR data (Table 1) indicated that **2** had a similar structure to that of **1**, except that the hydroxy at C-10 in **1** was reduced into methylene (*δ*_C_ 25.3; *δ*_H_ 2.88) in **2**, as supported by the HMBC correlations from *δ*_H_ 2.88 (2H, m, H-10) to *δ*_C_ 124.6 (s, C-9) and 65.4 (t, C-11), and the ^1^H‒^1^H COSY cross peak between H-10 and H-11. A product obtained by the chemical conversion from saponaceolide B showed NMR patterns identical to those of **2**, elucidating the absolute configuration of **2** to be the same to that of **1** (Scheme 1).

Compounds **1** and **2** were evaluated for their cytotoxicity to human MCF-7 and Hela cell lines. As a result, both of them possessed cytotoxic to two cell lines (Table 2). Significantly, compound **1** exhibited a strong inhibition on MCF-7 cell, with an IC_50_ value of 4.7 μM.

**Table 2:** Cytotoxicity of **1** and **2** (IC_50_, *μ*M)

Compd	MCF-7	Hela
**1**	4.7 ± 0.10	9.7 ± 0.08
**2**	13.9 ± 0.17	17.7 ± 0.13
Taxol^a^	< 0.008	< 0.008

^a^positive control

**Fig. 4:**
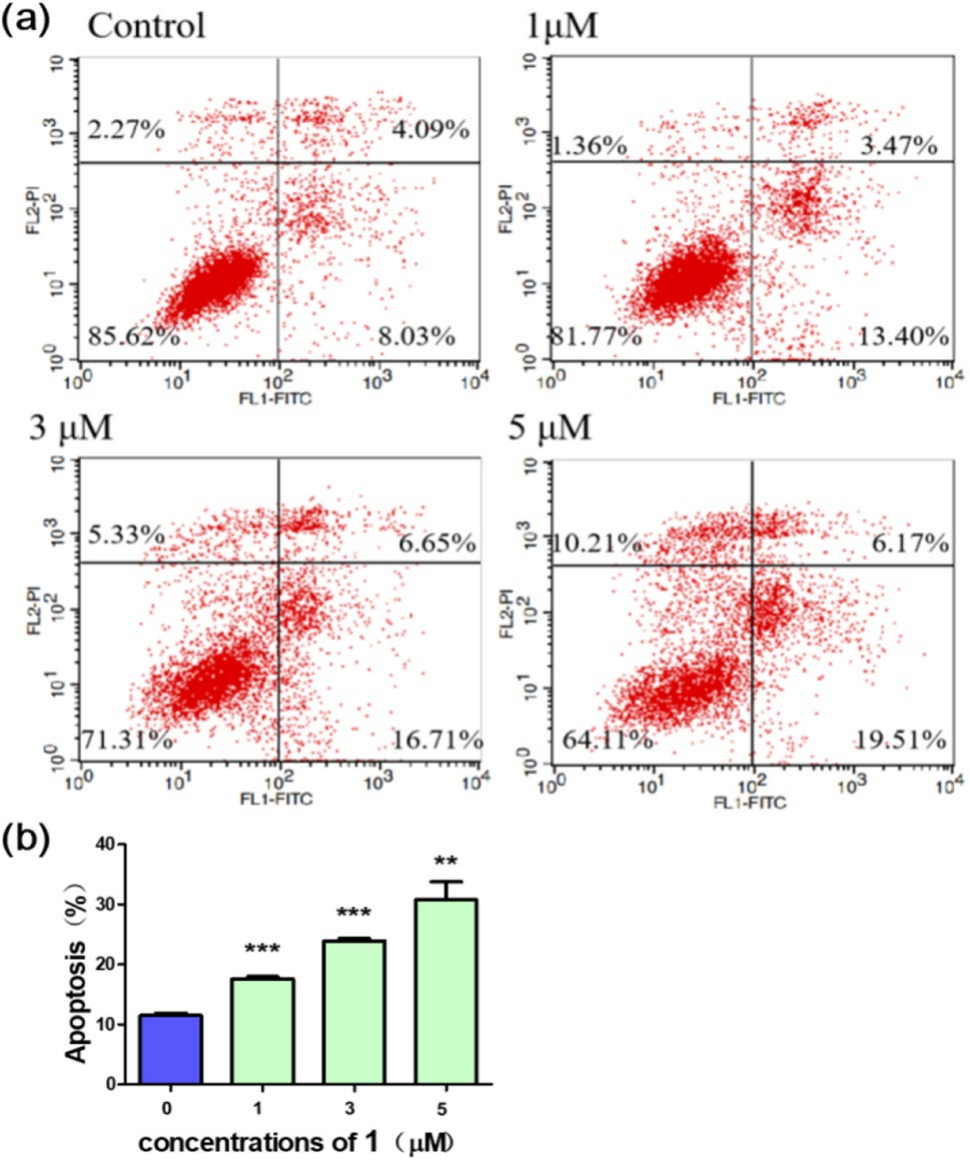
Effect of **1** on apoptosis of MCF-7 cells. (**a**) Induction of apoptosis was measured by annexin V/PI double-staining assay after treated with various concentrations (0 μM, 1 μM, 3 μM, 5 μM) of **1** for 24 h. (**b**) Apoptosis was expressed as a percentage of control. Data are presented as mean ± SD of triplicates. (*P < 0.05, **P < 0.01, ***P < 0.001 compared with control, n = 3)

Induction of apoptosis is considered as a possible mechanism of most of the chemotherapeutic agents, and targeting the apoptotic signaling system is becoming a promising strategy for the development of novel chemotherapeutic molecules [15]. In order to understand whether **1** inhibited cell growth through apoptosis, an annexin V/propidium iodide (PI) staining assay was used to detect the apoptotic rati [16]. As shown in Fig. 4, after treatment with 0 μM, 1 μM, 3 μM, and 5 μM of **1** for 24 h, the apoptosis rate increases gradually in a concentration-dependent manner. When drug concentration is 5 μM, the early apoptotic cells represent 19.51% of the total cells, which increases 11.48% than untreated cells. Meanwhile, the late apoptotic and necrotic cells represent 6.17% of the total cells, which increases 2.08% than untreated cells.

**Fig. 5:**
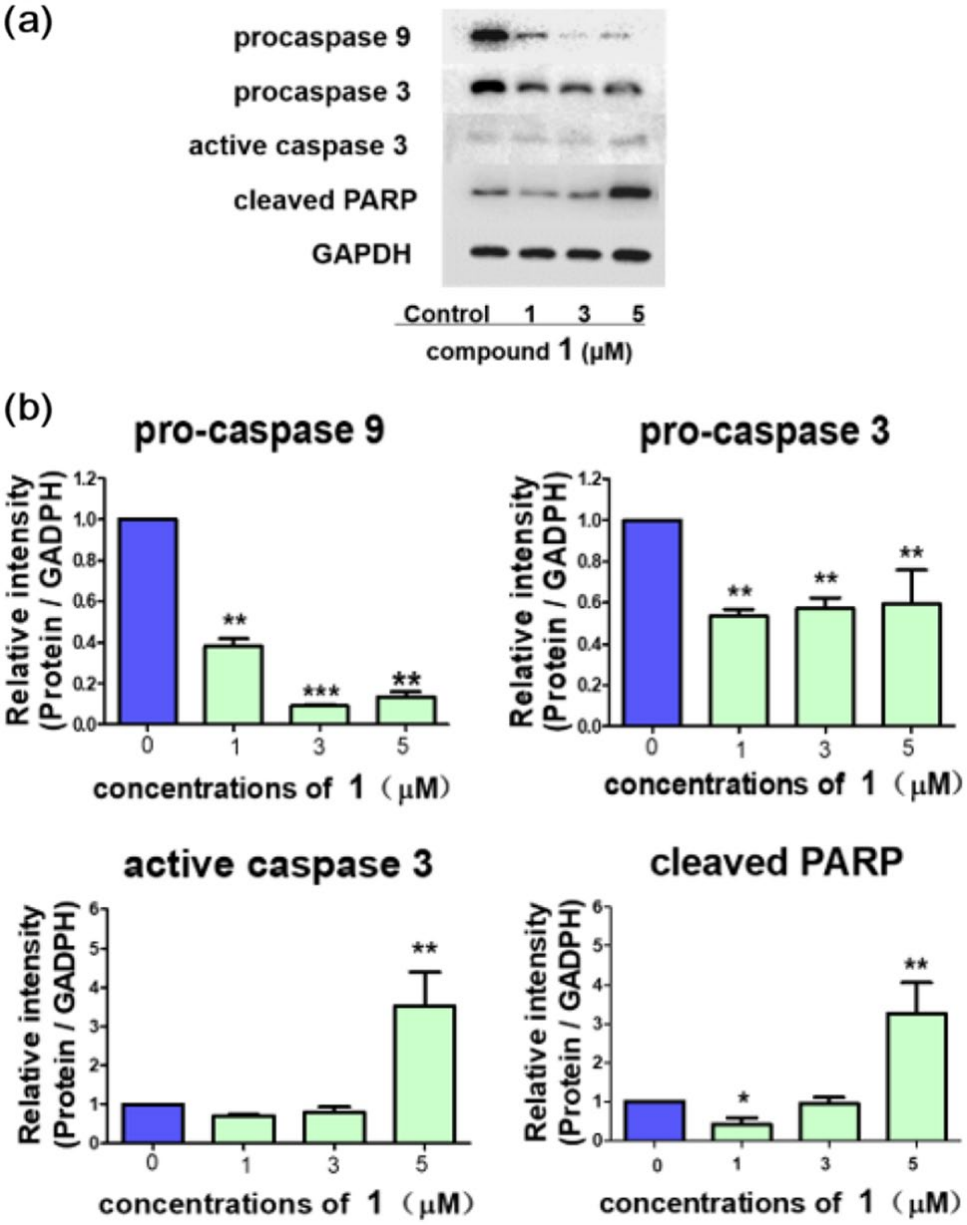
Effect of **1** on apoptotic relating-protein of MCF-7 cells. (**a**) Expression of apoptosis-related proteins in MCF-7 cells treated with various concentrations (0 μM, 1 μM, 3 μM, 5 μM) of **1** for 24 h. The levels of apoptotic proteins including caspase 3, caspase 9, and cleaved-poly-ADP-ribose polymerase (cleaved-PARP) were assessed by Western Blot assay. (**b**) The densitometric analysis represents the relative ratios of respective proteins to GAPDH. Data are presented as mean ± SD of triplicates. (*P < 0.05, **P < 0.01, ***P < 0.001 compared with control, n = 3)

Since the generation of cleaved poly-ADP-ribose polymerase (PARP) protein mediated by caspase family is considered as an important biomarker of apoptosis [17], the involvement of caspases in **1**-induced apoptosis was further examined. As shown in Fig. 5, caspase-3 and caspase-9 were both activated by comparing with the control groups. Then, the cleaved caspase-3 activated the self-cleavage of PARP, which gave rise to apoptosis. These results revealed that **1** could induce tumor cell death by apoptosis.

## Conclusions

In summary, two novel *Tricholoma* triterpenoids, namely tricholopardins C (**1**) and D (**2**), have been obtained from the fruiting bodies of the wild mushroom *T. pardinum*. The structures were unambiguously determined by analysis of their NMR and HRESIMS data, with the absolute configuration being confirmed by single-crystal X-ray diffraction. They possess a novel framework which was available by chemical conversions from saponaceolides A and B. The new modification of the isolated compound expand the chemical diversity of the *Tricholoma* triterpenoids family of natural products. Compound **1** exhibits significant cytotoxicity to MCF-7 by inducing apoptosis, presenting us with a great opportunity to discover promising natural agents for new antitumor drugs.

## Experimental Section

### General Experimental Procedures

Melting points were obtained on an X-4 micro melting point apparatus. Optical rotations were measured with a Horiba SEPA-300 polarimeter. IR spectra were obtained with a Tenor 27 spectrophotometer using KBr pellets. 1D and 2D spectra were run on a Bruker Avance III 600 MHz spectrometer with TMS as an internal standard. Chemical shifts (*δ*) were expressed in ppm with reference to the solvent signals. Mass spectra were recorded on an Agilent 6200 Q-TOF MS system. Column chromatography (CC) was performed on silica gel (200–300 mesh, Qingdao Marine Chemical Ltd., Qingdao, People’s Republic of China), RP-18 gel (20–45 µm, Fuji Silysia Chemical Ltd., Japan), and Sephadex LH-20 (Pharmacia Fine Chemical Co., Ltd., Sweden). Medium Pressure Liquid Chromatography (MPLC) was performed on a Büchi Sepacore System equipping with pump manager C-615, pump modules C-605 and fraction collector C-660 (Büchi Labortechnik AG, Flawil, Switzerland), and columns packed with RP-18 gel. Preparative High Performance Liquid Chromatography (prep-HPLC) was performed on an Agilent 1260 liquid chromatography system equipped with Zorbax SB-C18 columns (5 μm, 9.4 mm × 150 mm or 21.2 mm × 150 mm) and a DAD detector. Fractions were monitored by TLC (GF 254, Qingdao Haiyang Chemical Co., Ltd. Qingdao), and spots were visualized by heating silica gel plates sprayed with 10% H_2_SO_4_ in EtOH.

### Fungi Material

Wild mushrooms, *Tricholoma pardinum*, were collected from Schwarzwald in southwestern Germany in October 2014 and identified by Prof. Yu-Cheng Dai of Beijing Forestry University. A voucher specimen (No. HPCF20141008.6) was deposited at School of Pharmaceutical Sciences, South-Central University for Nationalities.

### Extract and Isolation

The air-dried fruiting bodies of *T. Pardinum* (1.5 kg) were extracted with MeOH (24 h × 3), and then partitioned with H_2_O and EtOAc (1:1). Finally, an EtOAc extract (56 g) was obtained, which was submitted to silica gel CC using CHCl_3_-MeOH (from 1:0 to 0:1) to give eight fractions (A-H). Fraction D (2.3 g) was separated by silica gel CC using petroleum ether-acetone (10:1‒4:1, v/v) to afford five sub-fractions (D1-D5). Fraction D4 (18 mg) was purified by HPLC (MeCN-H_2_O from 80:20 to 95:5, v/v, 25 min) to afford compound **2** (2.6 mg). Fraction E (4.8 mg) was separated by MPLC equipped with a RP-18 column eluted with MeOH-H_2_O (from 60:40 to 90:10, v/v, 40 min) to give six-subfractions (E1-E6). Fraction E3 was purified by HPLC (MeCN-H_2_O from 70:30 to 90:10, v/v, 20 min) to afford compound **1** (1.2 mg).

### Spectroscopic Data of Compounds

#### Tricholopardin C (**1**)

Colorless crystals, mp 126–128 °C; [*α*]_D_^25^ + 26.6 (c 0.22, MeOH); IR (KBr) *ν*_max_ = 3432, 2931, 1736, 1631, 1026, 1191, 1017 cm^‒1^; UV (MeOH): *λ*_max_ (log *ε*): 225 (3.33), 202 (3.79) nm; ^1^H and ^13^C NMR data, see Table 1; Positive ion HRESIMS *m/z* 523.30298 [M + Na]^+^ (calcd for C_30_H_44_O_6_Na^+^, 523.30301).

#### Tricholopardin D (**2**)

Colorless oil; [*α*]_D_^25^ ‒ 10.8 (c 0.15, MeOH); IR (KBr) *ν*_max_ = 2930, 1734, 1631, 1027, 1192, 1018 cm^‒1^; UV (MeOH): *λ*_max_ (log *ε*): 223 (3.31), 201 (3.82) nm; ^1^H and ^13^C NMR data, see Table 1; Positive ion HRESIMS *m/z* 507.30801 [M + Na]^+^ (calcd for C_30_H_44_O_5_Na^+^, 507.30810).

#### Crystal Data for Tricholopardin C (**1**)

A light colorless Prism-like of C_30_H_44_O_6_, *M* = 500.65, approximate dimensions 0.054 mm × 0.232 mm × 0.700 mm, was used for the X-ray crystallographic analysis on the BRUKER D8 QUEST. The integration of the data using a monoclinic unit cell yielded a total of 34,067 reflections to a maximum θ angle of 79.39° (0.78 Å resolution), of which 5674 were independent (average redundancy 6.004, completeness = 99.5%, R_int_ = 6.77%, R_sig_ = 4.65%) and 5231 (92.19%) were greater than 2σ(F^2^). The final cell constants of a = 8.3155(10) Å, b = 6.1091(8) Å, c = 26.765(3) Å, α = 90.00°, β = 94.589(5)°, γ = 90.00°, V = 1355.3(3) Å^3^, T = 100 (2) K. Data were corrected for absorption effects using the Multi-Scan method (SADABS). The structure was solved and refined using the Bruker SHELXTL Software Package, using the space group P 1 21 1, with Z = 2. The final anisotropic full-matrix least-squares refinement on F^2^ with 397 variables converged at R1 = 4.62%, for the observed data and wR2 = 12.44% for all data. The goodness-of-fit was 1.092. The absolute configuration was determined by the Flack parameter = -0.01(10), which was determined using 2110 quotients [(I +)-(I-)]/[(I +) + (I-)]. CCDC: 1,907,789 (https://www.ccdc.cam.ac.uk).

### Chemical Conversations of **1** and **2**

Saponaceolides A (5 mg) and B (3.5 mg) were added to toluene and stirred at 200 rpm for 12 h at 120 °C, respectively. The two reactants were separated by HPLC to give compound **1** (3.5 mg, yield = 70%) using a MeCN-H_2_O solvent system from 70:30 to 90:10 (v/v) in 20 min and compound **2** (2.1 mg, yield = 60%) using a MeCN-H_2_O solvent system from 80:20 to 95:5 (v/v) in 25 min, respectively.

### Cytotoxicity and Apoptosis Assay

#### Materials

Compounds **1** and **2** were tested for their cytotoxicity to human MCF-7 and Hela cell lines using the MTT method. Both compounds were dissolved in DMSO at 10 mM and stored at 4℃. MTT was purchased from Sigma—Aldrich (St. Louis, USA). Rabbit poly-antibody caspase-9, cleaved caspase-3, caspase-3, cleaved PARP, and secondary antibody conjugated horseradish peroxidase were purchased from AB clonal (Wuhan, China). Dulbecco’s modified Eagle’s medium (DMEM) and fetal bovine serum were obtained from HyClone (Logan, USA). Annexin V-FITC/PI apoptosis detection kit was purchased from Beyotime. (Nanjing, China) All reagents and compounds were analytical grades and commercially available.

#### Cell Culture

Human breast adenocarcinoma cell line (MCF-7) and human cervical carcinoma cell line (Hela) were purchased from Conservation Genetics CAS Kunming Cell Bank. The cells were cultured in high-glucose DMEM with 10% fetal bovine serum, 100 U/mL penicillin and 100 mg/mL streptomycin at 37 ℃ under an atmosphere of 5% CO _2_.

#### Cell Viability Analysis

Cell viability was assessed by the MTT assay. Cells were plated in 96-well plates at a density of 5000 cells in 200 μL of medium per well and incubated 4 h. The cells were treated with different concentrations of **1** and **2** for 24 h. At the end of the treatment, MTT solution (0.5 mg/mL in DMEM) was added and further incubated for 4 h. The absorbance was subsequently measured at 570 nm. Cell viability was calculated using the following formula: Cell viability % = (A_s_/A_0_) × 100%. A_s_ and A_0_ are the absorbance of the test substances and control, respectively. IC_50_ value represents the half of maximal inhibitory concentration.

#### Cell Apoptosis Analysis

MCF-7 cells were planted on 6-well plates and incubated overnight. Next, the cells were treated with different concentrations of **1** for 24 h. For flow cytometry, 1 × 10^6^ cells in 195 μL of binding buffer were stained with 5 μL of annexin V-FITC and 10 μL of PI at room temperature in the dark for 20 min. Cell apoptosis was analyzed using the BD Accuri ™ C6 flow cytometer.

#### Western Blot Analysis

MCF-7 cells were lysised with western blotting lysis buffer and 12000 g centrifugation for 10 min. The protein content of the supernatant was quantified using the BCA assay. Equal amounts of protein were separated on sodium dodecylsulfate polyacrylamide gels (SDS-PAGE) and electroblotted onto polyvinylidene difluoride (PVDF) membranes. Then, the membranes were blocked with blocking buffer (TBST with 5% skimmed milk) and incubated with primary antibodies overnight at 4 ℃. The membranes were washed with TBST and probed with secondary antibodies. Bound immunocomplexes were detected using a ChemiDOC TM XRS + system (Bio-Rad).

#### Statistical Analysis

All experiments were conducted more than three times independently. The results were analyzed by Tukey’s range test. The data are given as the mean ± standard error. P values < 0.05 were considered statistically significant.

## References

[1] Bray F, Ferlay J, Soerjomataram I, Siegel RL, Torre LA, Jemal A (2018). CA-Cancer J. Clin..

[2] Salas-Vega S, Iliopoulos O, Mossialos E (2017). JAMA Oncol..

[3] Newman DJ, Cragg GM (2020). J. Nat. Prod..

[4] G. F. Bills, J. B. Gloer, **4** (2016). 10.1128/microbiolspec.FUNK-0009-2016.

[5] Feng T, Gan XQ, Zhao YL, Zhang SB, Chen HP, He J, Zheng YS, Sun H, Huang R, Li ZH, Liu JK (2019). J. Nat. Prod..

[6] De Bernardi M, Garlaschelli L, Gatti G, Vidari G, Vita-Finzi P (1988). Tetrahedron.

[7] De Bernardi M, Garlaschelli L, Toma L, Vidari G, Vita-Finzi P (1991). Tetrahedron.

[8] Gozzini D, Mellerio GG, Gilardoni G, Clericuzio M, Vidari G (2018). Nat. Prod. Commun..

[9] Ovenden SPB, Yu J, Bernays J, Wan SS, Christophidis LJ, Sberna G, Tait RM, Wildman HG, Lebeller D, Platel D, May TW, Meurer-Grimes BM (2005). J. Nat. Prod..

[10] Yin X, Feng T, Shang JH, Zhao YL, Wang F, Li ZH, Dong ZJ, Luo XD, Liu JK (2014). Chem. Eur. J..

[11] Feng T, He J, Ai HL, Huang R, Li ZH, Liu JK (2015). Nat. Prod. Bioprospect..

[12] Zhao ZZ, Chen HP, Wu B, Zhang L, Li ZH, Feng T, Liu JK (2017). J. Org. Chem..

[13] Zhang SB, Li ZH, Stadler M, Chen HP, Huang Y, Gan XQ, Feng T, Liu JK (2018). Phytochemistry.

[14] Zhang FL, Yang HX, Wu X, Li JY, Wang SQ, He J, Li ZH, Feng T, Liu JK (2020). Phytochemistry.

[15] Ciriello G, Gatza ML, Beck AH, Wilkerson MD, Rhie SK, Pastore A, Zhang H, McLellan M, Yau C, Kandoth C, Bowlby R, Shen H, Hayat S, Fieldhouse R, Lester SC, Tse GMK, Factor RE, Collins LC, Allison KH, Chen Y-Y, Jensen K, Johnson NB, Oesterreich S, Mills GB, Cherniack AD, Robertson G, Benz C, Sander C, Laird PW, Hoadley KA, King TA, Perou CM, TCGA Research Network (2015). Cell.

[16] Panza E, Tersigni M, Iorizzi M, Zollo F, De Marino S, Festa C, Napolitano M, Castello G, Ialenti A, Ianaro A (2011). J. Nat. Prod..

[17] Green D, Kroemer G (1998). Trends Cell Biol..

